# Virulent nontyphoidal *Salmonella* producing CTX-M and CMY-2 β-lactamases from livestock, food and human infection, Brazil

**DOI:** 10.1080/21505594.2017.1279779

**Published:** 2017-02-16

**Authors:** Quézia Moura, Miriam R. Fernandes, Ketrin C. Silva, Daniel F. Monte, Fernanda Esposito, Milena Dropa, César Noronha, Andrea M. Moreno, Mariza Landgraf, Fábio J. Negrão, Nilton Lincopan

**Affiliations:** a Department of Microbiology, Institute of Biomedical Sciences, Universidade de São Paulo, São Paulo, Brazil; b Department of Clinical Analysis, School of Pharmacy, Universidade de São Paulo, São Paulo, Brazil; c School of Veterinary Medicine, Universidade de São Paulo, São Paulo, Brazil; d Food and Experimental Nutrition Department, School of Pharmacy & Food Research Center, Universidade de São Paulo, São Paulo, Brazil; e Public Health Laboratory, School of Public Health, Universidade de São Paulo, São Paulo, Brazil; f State Center for Clinical Analysis, São Paulo, São Paulo, Brazil; g Health Sciences Research Laboratory, School of Health Sciences, Universidade Federal da Grande Dourados, Dourados, Brazil

**Keywords:** ESBL, Food-producing animals, *Galleria mellonella*, IncI1, MLST, pAmpC, *Salmonella enterica*, Virulence genes

## To the Editor

Nontyphoidal *Salmonella enterica* (NTS) is a leading cause of bacterial foodborne disease throughout the world, and it is estimated to cause 93.8 million cases of gastroenteritis and 155,000 deaths each year.^1^ Although NTS generally causes self-limiting gastroenteritis, severe invasive infections can occur, requiring appropriate antimicrobial treatment.^2^ In this regard, the emergence of strains resistant to third-generation cephalosporins raises particular concern, since they are frequently chosen for the treatment of salmonellosis, mainly for invasive NTS infections.^3^ This resistance profile has been mostly attributed to the large dissemination of plasmids carrying genes encoding extended-spectrum β-lactamases (ESBLs) and plasmid-mediated AmpC β-lactamases (pAmpCs).^4^

Besides antibiotic resistance, virulence plays an important role in NTS infections, and the association of ESBL/pAmpC and a virulent profile in NTS strains represents a serious public health issue, once it makes these strains more harmful, contributing to the increase of morbidity and mortality rates.^5^ Moreover, it causes important economic impact due to medical costs, and losses on productivity and marketing of foods of animal origin.^6,7^

As the most populous country in South America, with more than 200 million inhabitants, Brazil has faced problems in controlling foodborne diseases. In this regard, between 2000 and 2015, according to data available by the Brazilian Ministry of Health, *Salmonella* was considered the major causative agent of foodborne outbreaks (http://www.saude.gov.br/svs). Because *Salmonella* is typically found in poultry, this type of meat has been an important vehicle of foodborne diseases. In fact, in Brazil, chicken meat is widely consumed, representing a potential risk to public health. So, the aim of the present study was to determine the genetic relatedness, the plasmid profile, and the virulence potential of ESBL/pAmpC-producing NTS strains from different serovars, sources and geographic locations in Brazil.

During a Brazilian multicentric antimicrobial resistance surveillance study, broad-spectrum cephalosporin resistance was investigated in 283 nontyphoidal *Salmonella* (NTS) isolates recovered from human (*n =* 4), farm animals (*n* = 2), food samples (*n* = 225) and other sources (*n* = 52), collected from 2008 to 2015. The isolates were identified by conventional biochemical methodology and, further, serotyped according to the Kauffmann-Le Minor scheme.^8^ Antimicrobial drug susceptibility was evaluated by disc diffusion method, according to the guidelines of the Clinical Laboratory Standards Institute.^9^

In this regard, from the 283 NTS investigated, ten isolates belonging to serovars Minnesota (*n* = 3, chicken meat), Typhimurium (*n* = 1, chicken meat), Infantis (*n* = 1, chicken meat), Heidelberg (*n* = 1, turkey meat), Agona (*n* = 1, turkey meat), Schwarzengrund (*n* = 1, drag swab), Muenchen (*n* = 1, human cerebrospinal fluid), and *S. enterica* subsp. *enterica* 4,5,12:i:- (*n* = 1, swine feces), exhibited resistance to cephalosporins, and were evaluated for the presence of ESBL, by the double-disc synergy test, and pAmpC, by resistance to cefoxitin.^9^ Further, individual polymerase chain reactions (PCR) were carried out to evaluate the presence of ESBL^10^ and pAmpC^11^ genes, and then, *bla*_CTX-M-8_ (*n* = 5), *bla*_CTX-M-2_ (*n* = 4), and *bla*_CMY-2_ (*n* = 1) genes were confirmed by sequencing, among different NTS serovars (Table 1).

**Table 1.** Epidemiologic and molecular characterization of ESBL/pAmpC-producing non-typhoidal *Salmonella* strains recovered from livestock, food and human infection, Brazil (2008–2015).

Strain	Serovar	Source	Year/State^a^	Resistance pattern^b^	MLST ST (CC)	ESBL/ pAmpC	Plasmid transfer^c^	Plasmid size (∼kb)	PBRT^d^	pMLST ST (CC)	Virulence genes	*Galleria mellonella* mortality (%)^e^
SAL785	*S.* Schwarzengrund	Drag swab	2008/PR	AMP, SAM, CRO, CTF, CIP, ENO, TET	96 (33)	CTX-M-2	T	291	UT	—	*aceK, invA, slyA, sopB*	100
SAL769	*S.* Agona	Turkey meat	2008/SC	AMP, SAM, CRO, CTF, CIP, ENO, TET, SXT	13 (54)	CTX-M-2	C	97	IncI1	113	*aceK, invA, slyA, sopB*	100
SAL58370	*S.* Typhimurium	Chicken meat	2010/MS	AMP, SAM, CRO, CTF, TET, SXT	19 (1)	CTX-M-2	T	240	UT	—	*aceK, h-1i, invA, slyA, sopB*	100
SAL65505	*S.* Minnesota	Chicken meat	2010/MS	AMP, SAM, CRO, CTF, TET, SXT	3088	CMY-2	C	97	IncI1	12 (12)	*aceK, invA, slyA, sopB*	100
SAL77088	*S.* Minnesota	Chicken meat	2010/PR	AMP, CRO, CTF	548 (77)	CTX-M-8	C	97	IncI1	113	*aceK, invA, slyA, sopB*	100
SAL68375	*S.* Heidelberg	Turkey meat	2010/MG	AMP, CRO, CTF	19 (1)	CTX-M-8	C	97	IncI1	113	*aceK, invA, slyA, sopB*	90
SAL70447	*S.* Minnesota	Chicken meat	2010/PR	AMP, CRO, CTF, TET	548 (77)	CTX-M-8	T	97	IncI1	113	*aceK, invA, slyA, sopB*	100
SAL219	*S. enterica* subsp *enterica* 4,5,12:i:-	Swine feces	2012/MG	AMP, SAM, CRO, CTF, CHL, CIP, ENO, TET, SXT	19 (1)	CTX-M-8	NT	340/97	IncP/UT	—	*aceK, h-1i, invA, slyA, sopB*	100
SAL14	*S.* Muenchen	Human infection	2013/SP	AMP, SAM, CRO, CTF, CIP, ENO, TET	112 (8)	CTX-M-2	NT	194	IncP	—	*aceK, invA, slyA, sopB*	100
SAL64	*S.* Infantis	Chicken meat	2015/SC	AMP, CRO, CTF, CHL, TET, SXT	32 (31)	CTX-M-8	C	97	IncI1	113	*aceK, invA, slyA, sopB*	100

Notes.

a PR, Paraná (South Brazil); SC, Santa Catarina (South); MS, Mato Grosso do Sul (Central-west); MG, Minas Gerais (South-east); SP, São Paulo (South-east).

b AMP, ampicillin; SAM, ampicillin-sulbactam; CRO, ceftriaxone; CTF, ceftiofur; CHL, chloramphenicol; CIP, ciprofloxacin; ENO, enrofloxacin; TET, tetracycline; SXT, trimethoprim/sulphamethoxazole.

c T, transformation; C, conjugation; NT, not transferred.

d UT, untyped.

e *G. mellonella* larvae were inoculated with 10^5^ CFU of NTS. SAL58370 and SAL219 caused 100% of mortality at 7 h post-infection; SAL70447 caused 100% of mortality at 8 h post-infection; SAL785, SAL769, SAL65505, SAL77088, SAL14, and SAL64 caused 100% of mortality at 21 h post-infection; SAL68375 caused 90% of mortality at 7 h post-infection; *S*. Typhimurium ATCC 14028 caused 40% and 60% of mortality at 7 h and 48 h post-infection, respectively; no mortality was observed in *G. mellonella* groups infected with ESBL-negative *S.* Typhimurium IAL 1431 and *E. coli* ATCC 25922 strains.

Multilocus sequence typing (MLST) was performed according to the *Salmonella enterica* MLST database (http://mlst.warwick.ac.uk/mlst/dbs/Senterica), and seven sequence types (STs) (Table 1) were identified, including a new ST. Most STs match with their respective serovars, with the exception of ST19, which was shared by *S*. Typhimurium, *S*. Heidelberg, and *Salmonella enterica* subsp *enterica* 4,5,12:i:-, suggesting that the expression of cell-surface antigens can be altered by horizontal transfer of genes and genetic recombination of chromosome, without interfering in the seven housekeeping genes used in the MLST scheme.^12^ The novel ST (ST3088) was detected in a *S*. Minnesota strain recovered from a chicken meat sample. According to data available in the *Salmonella enterica* MLST database (http://mlst.warwick.ac.uk/mlst/dbs/Senterica/GetTableInfo_html), most STs reported in this study correspond to the more common ones to their respective serovars, with a wide host range distribution around the world. However, with exception of *S*. Infantis ST32, the other six STs (i.e., ST13, ST19, ST96, ST112, ST548 and ST3088) have never before been reported in Brazil.^13^ Moreover, the human strain identified in this study corresponds to the first description (to our knowledge) of a CTX-M-2-producing *S*. Muenchen ST112 isolated from a cerebrospinal fluid sample of an infected newborn (Table 1).

Most plasmids carrying ESBL/pAmpC genes were successfully transferred by conjugation or transformation to *E. coli* J53, *E. coli* HB101 or *E. coli* TOP10 recipient strains. The size of plasmids carrying CTX-M- or CMY-2-type genes was estimated by S1 nuclease digestion following pulsed-field gel electrophoresis (S1-PFGE),^10^ ranging from ∼97 to 291-kb. Moreover, PCR-based replicon typing (PBRT)^14^ revealed that most plasmids harboring *bla*_ESBL_ and *bla*_CMY-2_ genes belonged to the IncI1 incompatibility group. IncI1 plasmids were submitted to plasmid multilocus sequence typing (pMLST) (http://pubmlst.org/plasmid/), most of them being assigned to ST113, with the exception of the *bla*_CMY-2_-carrying plasmid identified in the *S*. Minnesota strain from chicken meat, which belonged to ST12 (Table 1).

IncI1/ST113 plasmids harbouring *bla*_CTX-M-8_ gene have been previously reported in *Enterobacteriaceae* isolated from humans and food, in Germany.^15^ In Brazil, IncI1/ST113 plasmids have been associated with the transfer of *bla*_CTX-M-8_ gene in *E. coli* strains isolated from poultry.^16^ On the other hand, IncI1/ST12 plasmids have been disseminated worldwide, being linked to the spread of *bla*_CMY–_type pAmpC genes among members of the *Enterobacteriaceae* family from different sources and clinical contexts.^17,18^ Interestingly, in a recent study conducted in the Netherlands, IncI1/ST12 plasmids encoding *bla*_CMY-2_ were found in *S*. Heidelberg strains isolated from poultry meat imported from Brazil.^19^ So, our results support that IncI1/ST113 and IncI1/ST12 plasmids might be key vectors responsible for the dissemination of ESBL and pAmpC genes among NTS isolates through the Brazilian food production chain, which is worrisome, since Brazil is the third largest producer of chicken meat (only after the United States and China) and is the largest exporter of this product.^20^

Investigation of the virulence behavior of extended-spectrum cephalosporin-resistant NTS was initially performed using PCR for the detection of the chromosomal virulence genes *aceK*,^21^ *h-1i*,^21^ *invA*,^22^ *slyA*,^23^ and *sopB*,^21^ and the plasmidial virulence gene *spvC*,^22^ revealing the presence of *aceK, invA, slyA*, and *sopB* genes in all strains. In addition, *S*. Typhimurium and *S. enterica* subsp *enterica* 4,5,12:i:- strains, isolated from poultry meat and commercial swine, carried the *h-1i* gene (Table 1). The high similarity of virulence profile among different serovars of NTS from unrelated sources and geographic area, denote that important virulence genes might be highly conserved in NTS strains.^24^

In order to overcome the limitation related to the few virulence genes screened by PCR and the lack of their expression, which is necessary to demonstrate the virulence potential of strains, *in vivo* experiments were carried out with the *Galleria mellonella* infection model,^25,26^ using ESBL-negative *S.* Typhimurium ATCC® 14028™, *E. coli* ATCC® 25922™, and *S.* Typhimurium IAL 1431, as comparative strains. In this regard, *S.* Typhimurium IAL 1431, a drug-susceptible strain belonging to the culture collection of the National Reference Center Instituto Adolfo Lutz (São Paulo, Brazil), was negative for *aceK, invA* and *spvC* virulence genes. *G. mellonella* larvae, of nearly 250 to 350 mg, were inoculated with 10^5^ CFU of each strain and survival analysis was evaluated every hour, during 48 hours. For each strain, groups of *G. mellonella* containing five larvae were evaluated in two separate experiments.

**Figure 1.**
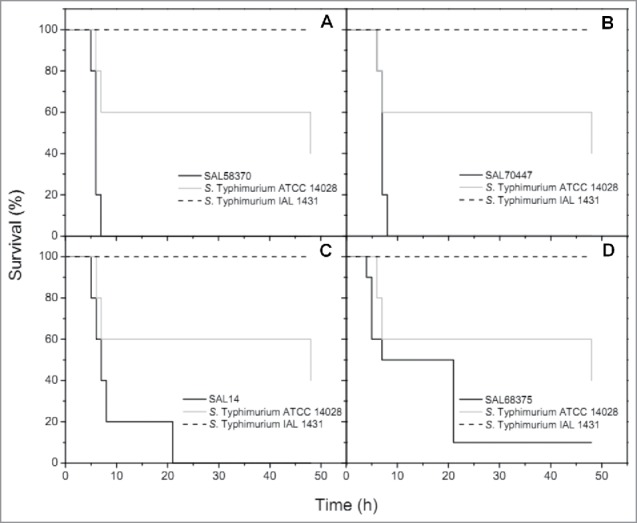
Kaplan-Meier survival curves of *Galleria mellonella* infected with 10^5^ CFU/larva of NTS strains. Cephalosporin-sensitive (ESBL-negative) *S.* Typhimurium ATCC 14028 and *S.* Typhimurium IAL 1431 were utilized as control strains. (A) Strain SAL58370, which had 100% mortality at 7 h post-infection. (B) Strain SAL70447, which had 100% mortality at 8 h post-infection. (C) Strain SAL14, which had 100% mortality at 21 h post-infection. (D) Strain SAL68375, which had 90% mortality at 7 h post-infection. Injection with the wild-type strains resulted in significantly higher mortality rate compared to injection with the control strains (*P*<0.05, log rank test).

Groups infected with *S.* Typhimurium SAL58370 and *S. enterica* subsp *enterica* 4,5,12:i:- SAL219 achieved 100% of mortality at 7 h post-infection, while a group infected with the *S.* Minnesota strain SAL70447, killed all the larvae at 8 h post-infection. For the *G. mellonella* groups infected with the other two *S.* Minnesota strains (SAL65505 and SAL77088) or with *S.* Schwarzengrund SAL785, *S.* Agona SAL769, *S.* Muenchen SAL14, or *S.* Infantis SAL64, 100% of mortality was achieved at 21 h post-infection. On the other hand, in the group infected with *S.* Heidelberg SAL68375, 90% of mortality was observed at 7 h post-infection (Table 1). No mortality was observed in larvae infected with *E. coli* ATCC 25922 and *S.* Typhimurium IAL 1431. Otherwise, *S*. Typhimurium ATCC 14028 killed 40% and 60% of the larvae at 7 h and 48 h post-infection, respectively. The Fig. 1 summarizes the *in vivo* evaluation of the virulence of four representative NTS strains in comparison to *S*. Typhimurium ATCC 14028 and *S.* Typhimurium IAL 1431. Survival curves were plotted using the Kaplan-Meier method, and data were analyzed by the log rank test, with *P*<0.05 indicating statistical significance (Graph Pad Software, San Diego, CA, USA). In this study, the low survival rates of *G. mellonella* suggest a high virulent background of NTS producing CTX-M and CMY-2 β-lactamases. In fact, mortality (%) of *G. mellonella* larvae infected with these strains was higher than in the ones infected with *S.* Typhimurium ATCC 14028, which is known to be highly virulent.^27,28^ Despite *G. mellonella* not being a natural host of *S. enterica*, it has been successfully utilized as an infection model to assess the pathogenic potential of NTS strains, since it displays many similarities with vertebrates, such as the innate immune system.^24,28,29^ Therefore, responses to bacterial infections observed in this model could closely mimics responses displayed by mammalian models.^30-32^ However, since *G. mellonella* infection model is not yet an established approach for the study of NTS, it not discards the need of using other models.

In summary, this study reports the emergence of virulent ESBL/pAmpC-producing NTS strains in food, food-producing animals and human over a 6-year period, in Brazil. The identification of IncI1/ST113 and IncI1/ST12 plasmids highlights the important role of these vectors in the spreading of ESBL and pAmpC genes among NTS belonging to different clinically significant serovars. Furthermore, the association of an extended-spectrum cephalosporin-resistant profile with a high virulence background deserves special attention, since NTS are a leading cause of food-borne zoonoses, constituting a major public health concern worldwide. So, meticulous investigation of strains of this sort is necessary to prevent their dissemination.
